# Heartworm prevalence in dogs versus cats: Multiple diagnostic modalities provide new insights

**DOI:** 10.1016/j.vpoa.2020.100027

**Published:** 2020-06-12

**Authors:** Kellie M. Hays, Jessica Y. Rodriguez, Susan E. Little, Annette L. Litster, Kennedy K. Mwacalimba, Kellee D. Sundstrom, Deborah M. Amodie, Maria A. Serrano, Simone D. Guerios, Jennifer N. Lane, Julie K. Levy

**Affiliations:** a Maddie’s Shelter Medicine Program, Department of Small Animal Clinical Sciences, College of Veterinary Medicine, 2015 SW 16thAvenue, University of Florida, Gainesville, FL 32610, USA; b Zoetis Petcare, 10 Sylvan Way, Parsippany, NJ 07054, USA; c Department of Veterinary Pathobiology, Oklahoma State University, 250 McElroy Hall, Stillwater, OK 74078, USA; d Zoetis Outcomes Research, 10 Sylvan Way, Parsippany, NJ 07054, USA; e Department of Small Animal Clinical Sciences, College of Veterinary Medicine, 2015 SW 16th Avenue, University of Florida, Gainesville, FL 32610, USA

**Keywords:** Animal shelter, Cat, *Dirofilaria immitis*, Dog, Heartworm, Heartworm test/testing, Prevalence

## Abstract

The cornerstones of diagnosis of heartworm (HW) in dogs are the detection of circulating antigen from adult female *Dirofilaria immitis* or the visualization of microfilariae in whole blood. These tests are less sensitive in cats because of the feline immune response leading to low numbers of adult worms, but heartworm antibody tests are also licensed for use in cats. HW antibodies in cats are detectable when there has at least been larval development in the tissues, but positive antibody tests cannot distinguish between current and previous larval infections; thus, cats with positive antibody test results are considered currently or previously infected with *D. immitis*. The aim of the present study was to use multiple HW diagnostic modalities to maximize detection of infection in dogs and cats at high risk of infection and to compare infection prevalence between these two hosts. Blood samples collected from 100 stray dogs and 100 stray cats at Florida animal shelters were tested for HW antigen (before and after heat treatment) and microfilariae; cats were also tested for HW antibody. Dogs were significantly (*P* = 0.0001) more likely to be diagnosed with adult HW infection (28 %; 95 % CI: 20.1–37.6%) when compared with cats (4 %; 95 % CI: 1.6–10.2%) on the basis of positive antigen and microfilariae test results. Cats with current or previous adult, immature adult, or larval HW infections comprised 19 % (95 % CI: 12.4%–27.9%) of the feline population, which was not significantly different (*P* = 0.1) from the prevalence of adult *D. immitis* infection in dogs. Testing unprotected cats for heartworm antibodies demonstrated a similar, high risk of infection to the matched unprotected dog population in Florida, which supports the use of HW preventives in cats in areas where HW transmission occurs.

## Highlights

• Prevalence of adult heartworm (HW) infection was 4 % in cats and 28 % in dogs.
• Combining antigen and antibody testing led to an overall 19 % positive cats.
• Prevalence did not differ between dogs and cats with added feline antibody testing.
• *Dirofilaria repens* microfilariae were identified in one dog and one cat.
• *Acanthocheilonema reconditum* microfilariae were identified in four dogs.

## 1 Introduction

*Dirofilaria immitis*, commonly referred to as heartworm (HW), is an internal parasite of dogs and cats that causes pathology in the heart, pulmonary arteries, and lung parenchyma. Infective 3rd stage larvae are transmitted by mosquito vectors and develop through tissue phases into adult nematodes in the pulmonary arteries (Bowman and Atkins, 2009, Lee and Atkins, 2010). Adult *D. immitis* reside in the main pulmonary artery approximately 4 months after initial infection, with antigen detected 4.2 months after infection if samples are heated prior to testing, or 6–9 months after infection if samples are not pre-treated (Bowman and Atkins, 2009, Carmichael et al., 2016). Mature worms reproduce sexually and release microfilariae into the circulation 6–9 months post infection, serving as a source of infection for mosquitoes to complete the life cycle (Bowman and Atkins, 2009, Ledesma and Harrington, 2011). Dogs and some wild canids are the final/reservoir hosts, and while clinical signs do not always occur, canine clinical HW infections are characterized by cardiopulmonary signs such as coughing, exercise intolerance, and abnormal heart sounds (Bowman and Atkins, 2009). Over time, ascites secondary to pulmonary hypertension and right sided heart failure can occur (Bowman and Atkins, 2009). High intensity infections (Courtney and Zeng, 1989) can result in physical disruption to tricuspid valve function (caval syndrome) associated with acute weakness and hemoglobinuria (Strickland, 1998, Kaiser and Williams, 2004, Winter et al., 2017).

Feline infections can also be asymptomatic, but when clinical signs occur they can happen as early as three months post infection and are most commonly associated with the inflammatory responses in the pulmonary vasculature and parenchyma to immature adult infections, which can cause intermittent coughing, dyspnea, and wheezing (Lee and Atkins, 2010). When adult HW infection occurs in cats, low intensity infections are typical and worm death can precipitate an acute, shock-like reaction, resulting in the death of the cat with few, if any, premonitory signs (Litster et al., 2008). Treatment in cats is usually limited to suppressing the pulmonary inflammatory response using diminishing doses of glucocorticoids. There is no approved adulticidal therapy in cats; however, there are reported cases of surgical extraction of adult worms in cats leading to improvement of clinical signs (Lee and Atkins, 2010).

A number of testing modalities are available to identify HW infection in cats and dogs, each capable of detecting different stages of the life cycle. Antigen tests detect free, unbound antigen from gravid females; thus, low worm numbers and single-sex infections can result in false-negative antigen test results (Levy et al., 2003, Little et al., 2014, DiGangi et al., 2017, Gruntmeir et al., 2017, Little et al., 2018). In some infected dogs and many cats, antigen may become trapped in immune complexes, reducing free antigen to undetectable amounts and producing false-negative results (Little et al., 2014, Little et al., 2018). Low worm numbers and single-sex infections present similar challenges to microfilarial test sensitivity, especially in cats. While concentration techniques can be used to increase the sensitivity of detection of microfilariae in whole blood, morphological identification can be complicated by the similarity between microfilariae of *D. immitis* and those of *D. repens, Acanthocheilonema reconditum,* and *A. dracunculoides*. Antibody tests for cats primarily detect the humoral immune response to migrating third and fourth stage larvae in the pulmonary tissue (Snyder et al., 2000, Levy et al., 2003). A HW antibody positive test in a cat can represent several infection states: infection with early larval stages, immature worms, adult worms, or previous infection with any of these stages. Antibody testing is the only means of identifying immature infections in cats, and cats with clinical signs are more likely to test antibody-positive than asymptomatic cats (Lee and Atkins, 2010). Experimental infections have demonstrated that antibodies can persist for months after infection even if infections were abbreviated with a macrocyclic lactone 28 days after infection (Dillon et al., 2017). In one study that followed a small cohort of naturally infected cats identified by positive antibody tests, cats became antibody negative up to one to three years after initial detection (Venco et al., 2011). Therefore, positive antibody results cannot indicate when infection was acquired or if the cat is currently infected with immature or adult worms, especially in unprotected cats living in areas where infected mosquito vectors occur. Also, cats that are currently antibody negative could have been infected with HW in the past, and some antibody-negative cats harbor adult worms (Berdoulay et al., 2004).

While multiple epidemiologic surveys have separately reported regional or national prevalence of HW infection in dogs and cats (Levy et al., 2003, Bowman and Atkins, 2009, Tzipory et al., 2010, Venco et al., 2011, Little et al., 2014, Levy et al., 2017, Drake and Parrish, 2019, Self et al., 2019), we are unaware of reports comparing prevalence of infection in age-matched cats and dogs from the USA that were screened contemporaneously with a comprehensive panel of diagnostic tests. The aim of this study was to use multiple HW diagnostic modalities to maximize detection of HW infection in dogs and cats and compare prevalence between these two hosts. In this study we define overall prevalence of infection in dogs by evidence of adult heartworm infection (antigen and/or microfilariae positive). In cats we define overall prevalence of infection by evidence of current or previous infection with *D. immitis* larval or adult stages (positive antigen, microfilariae, and/or antibody test).

## 2 Materials and methods

### 2.1 Animals and samples

One hundred dogs and 100 cats in Florida animal shelters were selected for inclusion in the study. Selection criteria included estimated minimum age of 2 years, stray intake status, and no history of treatment with adulticidal or microfilaricidal medications. No animals had apparent clinical signs that could be attributed to heartworm disease and animals were not specifically selected for healthy status; however, strays recently admitted to the shelter may have had signs that went unnoticed by staff. To control for similar potential exposure time to HW infection, pairs of dogs and cats were matched within 2 years of estimated age so that the age distribution within each host group was similar. Information collected for each animal included identification number, species, sex, age (estimated by dentition by at least two members of the research team), and body weight. Samples were collected in May and June 2019, as part of each animal’s routine health examination. Residual samples were evaluated on an extended panel of HW diagnostic tests. This study was approved by the University of Florida Institutional Animal Care and Use Committee (Protocol Number: #201910605; Approval date: 02 February 2019).

Whole blood collected in serum-separator tubes was allowed to clot at room temperature (30 min) and then centrifuged for 10 min. Whole blood preserved in EDTA and serum samples were packed in insulated shipping boxes with ice packs and shipped by overnight courier to the Center for Veterinary Health Sciences at Oklahoma State University for further analysis.

### 2.2 Diagnostic testing

Diagnostic assays were performed by three different research teams, each masked to the others’ results. One team collected samples and performed the point-of-care testing at each shelter; a second team performed point-of-care and microtiter plate ELISA testing, point-of-care antibody testing, and submission of samples to a commercial laboratory for antibody tests; and a third group performed microfilariae PCR and analyzed sequence data obtained through an academic core facility.

#### 2.2.1 Antigen testing

Whole blood anticoagulated with EDTA was tested for *D. immitis* antigen at each shelter on the day of collection using a point-of-care assay (WITNESS® Heartworm Test Kit; Zoetis, Parsippany, NJ, USA). Feline blood samples were also tested for FeLV antigen and FIV antibodies at each shelter using a point-of-care assay (WITNESS® FeLV-FIV; Zoetis, Parsippany, NJ, USA). Serum was tested for HW antigen by a microtiter plate ELISA method (DiroCHEK® Heartworm Antigen Test Kit; Zoetis, Parsippany, NJ, USA) according to manufacturer’s instructions, using samples both before and after heat treatment. Heat treatment was performed by heating serum to 104 °C for 10 min, then centrifuging at 16,000 x *g* to separate the liquid phase to be used in the ELISA (Little et al., 2018). After performing these tests and assessing marked discordant canine results between the WITNESS® Heartworm Test Kit and the DiroCHEK post heat treatment, stored canine serum was tested for HW antigen by a second point-of-care assay (SNAP® Heartworm RT Test; IDEXX Laboratories, Inc., Westbrook, ME, USA) to further examine test comparison between the DiroCHEK post heat treatment and another point-of-care test (Table 1)

**Table 1:** Percentage of heartworm positive tests in 100 dogs and 100 cats recently admitted to Florida animal shelters as strays.

	WITNESS Ag	SNAP Ag	DiroCHEK Ag	DiroCHEK HT Ag	Modified Knott's *Di* Mf	Wet Mount *Di* Mf	Antech Ab	Solo Step Ab
	% Positive							
Dogs (*n* = 100)	14	14	16	28	13^a^	12	NP	NP
Cats (*n* = 100)	2^b^	NP	3^b^	3^b^	0^b^, ^c^	0^b^	2	17

NP, Not performed; Ag, Antigen; HT, Heat treatment; *Di*, *Dirofilaria immitis*; Mf, Microfilariae; Ab, Antibody.

a Microfilariae were identified in an additional five dogs (5%), but were identified as other filarial species by morphology and/or sequencing: *Acanthocheilonema reconditum* (*n* = 4); *Dirofilaria repens* (*n* = 1).

b The proportion of positive tests was significantly higher for dogs than for cats in each test method (*P* < 0.05). Antibody test results were combined in the comparative analysis.

c Microfilariae were identified in two cats (2%), but were identified as other filarial species by morphology and/or sequencing: *Dirofilaria repens* (*n* = 1); *Dirofilaria sp.* (*n* = 1).

#### 2.2.2 Microfilariae detection

Blood was refrigerated at 4 °C pending microfilariae testing within 4 days of collection. A wet mount and modified Knott’s test was performed on each sample. The wet mount was prepared by mixing 10 μL of blood with 10 μL of saline on a glass slide and placing a cover slip (22-mm square) over the mixture; the entire area under the coverslip was examined by microscopy for microfilariae using the 10x objective (100x magnification). The wet mount preparation was reported as positive if any microfilariae were observed, but specific identification was not attempted due to the inability to measure motile microfilariae length and width. The modified Knott’s test was performed as previously described (Zajac and Conboy, 2012). Briefly, 1 mL of whole blood was placed in a 15 mL centrifuge tube, 9 mL of 2 % formalin added, and the tube inverted several times to lyse the red blood cells. The mixture was centrifuged at 500 x *g* for 5 min, the supernatant decanted, and the pellet stained with 0.1 % methylene blue. The stained material was transferred to 2–4 microscope slides, a coverslip (24-mm x 60-mm) applied to each slide, and the entire pellet examined for microfilariae. For samples with a very large number of microfilariae evident on wet mount, the modified Knott’s test was performed on a smaller volume of blood (0.1−0.25 mL). All microfilariae present were counted, the number of microfilariae per mL calculated, the length and width of up to 10 microfilariae present in a sample recorded, and a preliminary morphologic identification made by comparison to published descriptions (Zajac and Conboy, 2012). Whole blood was held frozen at −20 °C for PCR testing to confirm the microfilariae species.

#### 2.2.3 Microfilariae PCR

Microfilariae were identified in frozen blood samples by PCR and sequencing as previously described (Otranto et al., 2011). Briefly, total nucleic acid was extracted from 200 μL of whole blood with a commercial kit (DNeasy Blood & Tissue Kit; Qiagen, Hilden, Germany) and used as template in a PCR targeting a ∼ 330 bp fragment of the 12S rRNA gene. Amplification was confirmed on a 2% agarose gel; amplicons were column-purified and sequenced directly with an ABI 3730 capillary sequencer (Applied Biosystems;Foster City, California, USA) at the Oklahoma State University Molecular Core Facility (Stillwater, Oklahoma, USA). Electropherograms were verified by visual inspection, compared with all available sequences in GenBank (National Center for Biotechnology Information), and the GenBank accession numbers of sequences with the closest identity reported (NCBI National Center for Biotechnology Information, 2019).

#### 2.2.4 Antibody detection

Feline serum was tested for HW antibodies using a point-of-care kit (Solo Step® FH; Heska, Loveland, CO, USA) according to manufacturer’s instructions. Feline serum was also tested in triplicate by a commercial laboratory (Heartworm Antibody Feline; Antech™ Diagnostics, Fountain Valley, CA, USA).

### 2.3 Statistical analysis

Dogs and cats were considered HW-infected if they had a positive result on any antigen test or microfilariae test (Courtney and Zeng, 2001, Atkins, 2003, Gruntmeir et al., 2017). Additionally, cats were considered to have current or previous larval infection if they had a positive antibody test result (Goodwin, 1998). Heartworm prevalence was defined as a binary variable (yes = 1; no = 0) and analyzed by a linear mixed model approach. Using the SAS Proc Glimmix procedure (SAS 9.4; Cary, NC), prevalence was analyzed with a model that considered the fixed effect of vertebrate host (cat or dog) and the random effects of shelter and the residual error. This analysis utilized a binomial error and logit link. The 5% level of significance (*P* < 0.05) was used to assess statistical differences.

Prevalence was compared by use of a χ2 test or Fisher’s exact test, as appropriate, and odds ratios with their 95 % confidence intervals were calculated (VassarStats: Statistical Computation Web Site, 2020) for species, sex, estimated age, weight, location, and presence of FeLV or FIV*. P* < 0.05 was considered to be statistically significant.

## 3 Results

### 3.1 Animals

A total of 100 dog-cat pairs matched within 2 years of age were enrolled in the study. Of these, 87 animals (dogs, *n* = 49; cats, *n* = 38) were presented to two shelters in Columbia County and Marion County (North-Central Florida), and 113 animals (dogs, *n* = 51; cats, *n* = 62) were presented to a shelter in Miami-Dade County (South Florida). The mean estimated age of dogs was 3.5 ± 0.2 years (median, 3 years; range, 2−12 years), and was not significantly different from the mean estimated age of cats (3.3 ± 0.2 years; median, 3 years; range 2−12 years; *P* = 0.5).

### 3.2 Diagnostic test results

Diagnostic test results are summarized in Table 1. Dogs were significantly (*P* = 0.0001) more likely to be diagnosed with adult HW infection (28 %) than cats (4 %; Table 2). When antibody testing was used in conjunction with antigen testing for feline HW infection, cats were more likely to be diagnosed with current or previous adult, immature worm, or larval infection (19 %) than adult HW infection only (4 %; *P* = 0.001; Table 2). The prevalence of positive HW antibody test results, indicating current or previous adult, immature worm, or larval infections in cats, was 17 %, and 19 % of cats were positive for antigen or antibody, which was not significantly different from the prevalence of adult HW infections in dogs (*P* = 0.2).

**Table 2:** Prevalence of heartworm (HW) infection in cats compared to dogs in Florida shelters.

Category	Tested (*n*)	Negative (*n*)	Positive (*n*)	% Positive
Cat adult HW infection (Ag or Mf)	100	96	4	4
Cat adult HW infection or previous/current larval HW infection (antigen, antibody, or Mf)	100	81	19	19
Dog adult HW infection (antigen or Mf)	100	72	28	28

Mf, microfilariae.

#### 3.2.1 Antigen test results

The prevalence of antigen-positive cats did not increase with heat treatment. Two out of the four positive antigen tests were positive both before and after heat treatment. Another cat was only antigen-positive before heat treatment and the other converted from antigen-negative to antigen-positive after heat treatment. Both cats that were antigen-positive before and after heat treatment were also antibody-positive. The other two cats did not have detectable antibodies.

In dogs, all 16 specimens with positive antigen test results before heat treatment were also positive after heat treatment. Another 12 antigen-negative specimens tested antigen-positive after heat treatment.

#### 3.2.2 Microfilariae detection

Microfilariae were identified by direct examination of wet mount preparations in 17 dogs and one cat; modified Knott’s test revealed microfilariae in one additional dog and one additional cat (Table 3). In microfilaremic dogs (*n* = 18), morphometric evaluation of microfilariae was consistent with *D. immitis* (*n* = 13), *A. reconditum* (*n* = 4), and a *Dirofilaria* sp. (*n* = 1). Sequencing of a 12S rDNA fragment confirmed all microfilariae species and identified the *Dirofilaria* sp. as *D. repens* (Table 3). In microfilaremic cats (*n* = 2), morphometric evaluation of microfilariae was consistent with *Dirofilaria* sp. (*n* = 2). Sequencing of a 12S rDNA fragment identified the *Dirofilaria* sp. as *D. repens* (*n* = 1) and an unknown *Dirofilaria* sp. (*n* = 1) (Table 3). Co-infections were not evident by morphometric evaluation of microfilariae or by sequencing. Of the 18 dogs with microfilariae, 13/18 (72 %) were positive for antigen before heat treatment and all (18/18; 100 %) were positive after heat treatment, regardless of microfilariae identity. One of the microfilariae-positive cats (*D. repens*) was positive for both antigen and antibodies, and one (unidentified *Dirofilaria* sp.) was positive for antibodies only.

**Table 3:** Microfilaria identified in stray dogs (*n* = 18) and cats (*n* = 2) from Florida, 2019.

Animal number	Wet mount	Modified Knott’s test, morphology	Modified Knott’s test (number/mL)	Sequence confirmation
D02	Positive	*Ar*	142	*Ar*
D14	Positive	*Di*	15,220	*Di*
D22	Positive	*Ar*	47	*Ar*
D24	Positive	*Ar*	7	*Ar*
D27	Positive	*Di*	3400	*Di*
D28	Positive	*Di*	58	*Di*
D31	Positive	*Di*	1102	*Di*
D32	Positive	*Di*	2050	*Di*
D34	Positive	*Ar*	3	NA
D43	Positive	*Di*	606	*Di*
D48	Negative	*Di*	13	*Di*
D53	Positive	*Di*	11,740	*Di*
D57	Positive	*D.* sp.	158	*Dr*
D81	Positive	*Di*	8210	*Di*
D83	Positive	*Di*	5250	*Di*
D87	Positive	*Di*	180	*Di*
D90	Positive	*Di*	43,280	*Di*
D99	Positive	*Di*	1293	*Di*
C56	Positive	*D.* sp.	1099	*Dr*
C95	Negative	*D.* sp.	3	*D.* sp.^a^

D, Dog; C, Cat; *Ar, Acanthocheilonema reconditum*; *Di, Dirofilaria immitis*; *Dr, Dirofilaria repens*; *D*. sp., *Dirofilaria* sp.; NA, No amplification.

Sequence confirmation by identity to GenBank Accession Numbers KF707482.1 (*D. immitis*), JF461460.1 (*A. reconditum*), or AM779775.1 (*D. repens*).

a Not consistent with any *Dirofilaria* sp. with sequence available in GenBank.

### 3.3 Risk factors for heartworm infection

In dogs, age was the only significant risk factor for HW infection (Table 4). The infection prevalence in dogs greater than 2 years old was significantly greater than in 2 year old dogs (OR = 3.5, 95 % CI = 1.3−9.6, *P* = 0.02). In cats, there were no significant risk factors identified for current or previous larval HW infection or current adult HW infection (*P* > 0.05; Table 5).

**Table 4:** Risk factors for heartworm (HW) infection in dogs from Florida shelters.

Factor	Category	Tested (*n*)	Negative (*n*)	Positive (*n*)	% Positive	Odds ratio	95 % CI	*P* value
Dog age	2 years	41	35	6	15	Reference		
	> 2 years	59	37	22	37	3.5	1.3−9.6	0.02
Deg sex	Female	46	36	10	22	Reference		
	Male	54	36	18	33	1.8	0.7−4.4	0.3
Dog weight	< 15 kg	13	12	1	8	Reference		
	15−25 kg	52	37	15	29	4.9	0.6−40.8	0.2
	> 25 kg	35	23	12	34	6.3	0.7−54.1	0.08
Dog region	North-Central Florida	49	33	16	33	Reference		
	South Florida	51	39	12	24	0.6	0.3−1.5	0.4

95 % CI, 95 % Confidence interval.

**Table 5:** Risk factors for HW antigen and/or antibody positive status in cats from Florida shelters.

Factor	Category	Tested (*n*)	Negative (*n*)	Positive (*n*)	% Positive	Odds ratio	95 % CI	*P* value
Age	2 years	42	33	9	21	Reference		
	> 2 years	58	48	10	17	0.8	0.3−2.1	0.6
Sex	Female	53	42	11	21	Reference		
	Male	47	39	8	17	0.8	0.3−2.1	0.8
Bodyweight	< 3.5 kg	49	39	10	20	Reference		
	≥ 3.5 kg	51	42	9	18	0.8	0.3−2.3	0.8
FeLV status	FeLV-negative	96	78	18	19	Reference		
	FeLV-positive	4	3	1	25	1.4	0.1−14.7	1
FIV status	FIV-negative	89	73	16	18	Reference		
	FIV-positive	11	8	3	27	1.7	0.4−7.2	0.7
Region	North-Central Florida	38	33	5	13	Reference		
	South Florida	62	48	14	23	1.9	0.6−5.9	0.3

95 % CI, 95 % Confidence interval; FeLV, Feline leukemia virus; FIV, Feline immunodeficiency virus.

## 4 Discussion

To the authors’ knowledge, this is the first study to compare HW infection prevalence in contemporaneously collected specimens from dogs and cats sourced from the same region in the United States, using a comprehensive array of testing modalities (pre and post heat treatment antigen, modified Knott’s with PCR and sequencing of microfilariae, and feline HW antibody testing). While evidence of the presence of adult worms in dogs was higher than that in cats, when evidence of current or previous adult, immature worm, or larval HW infection in cats was included, the prevalence in dogs and cats was not statistically different. This observation is consistent with the interpretation that while a relatively high percentage of cats are bitten by mosquitoes and infected with third-stage larvae, many larval infections do not progress to the adult stage. Because cats can exhibit respiratory disease from the robust inflammatory response to immature worms arriving in the pulmonary arteries as early as 3 months post infection, heartworm antigen prevalence studies in cats should not be used as a surrogate for risk of heartworm infection and heartworm disease in cats regardless of whether heat treatment is performed prior to antigen testing feline samples (Lee and Atkins, 2010, Little et al., 2018).

Studies in Europe have compared canine antigen prevalence with feline *D. immitis* antibodies and *Wolbachia* surface protein (WSP) antibodies. In Grand Canaria, Spain, the prevalence of antibodies in cats was statistically higher (33 %) compared to antigen testing in dogs (19 %) (Montoya-Alonso et al., 2011). In another study, in northern and central Portugal, the seroprevalence of feline HW infection was 15 % compared to 2.1 % HW antigen prevalence in dogs (Vieira et al., 2014). These studies differed from ours in that these were client-owned animals, dogs may have been on heartworm preventive, and dogs and cats were not age-matched. Heat treatment was also not used in antigen testing. In a shelter study, dogs that had previously received a heartworm preventive had a greater odds of antigen blocking than dogs that had not been on prevention, and intentional use of some preventives with doxycycline in infected dogs is associated with false negative antigen test results (Drake et al., 2015, DiGangi et al., 2017). If heat treatment had been performed in the earlier studies comparing infection in dogs and cats, it is possible that the comparative prevalence of heartworm infection could have been more similar. Another comparative study in Po River Valley, Italy tested dogs for antigen and microfilariae, but tested cats for antigen only if they initially tested positive for HW antibody (Venco et al., 2011). HW antibody prevalence in cats was 4.7 % compared to dogs that were a combined 29 % positive by modified Knott’s and antigen testing (Venco et al., 2011). Again, this study differed from ours in that these were client-owned animals, dogs could have been on heartworm preventive, and heat treatment was not used in antigen testing. In these types of comparative studies, it is important to note patient and test selection when interpreting results.

A shelter-based necropsy study conducted in northern Florida reported that prevalence of infection with adult worms in cats was 5%, prevalence of exposure (antibody positive) was 17 %, and that male cats in that population were more likely to be infected with HW (Levy et al., 2003). Further, 7 % of cats tested antigen-positive, and some cats identified as infected on necropsy had false-negative antigen test results. This emphasizes the need for a broad approach when testing cats for HW infection, using more than one test so that discordances can be identified and interpreted. Another Florida shelter study reported HW antigen-positive prevalence in cats of approximately 8 % and antibody-positive prevalence of approximately 23 % (Berdoulay et al., 2004). In a study that compared necropsy results with antibody test results, 6 % of cats had adult infections, but 14–26 % were antibody positive, depending on brand of antibody test used (Snyder et al., 2000). Our data also demonstrated that antibody-positive test results were more common than antigen-positive results in cats, suggesting a predominance of larval or immature worm infections rather than adult HW infections. However, it is known that when clinical signs of infection occur in cats, they are most commonly driven by the immune reaction to immature worms in the pulmonary arteries, resulting in intermittent coughing and dyspnea (Lee and Atkins, 2010). Increased awareness of the prevalence and potential effects of overlooked larval and immature HW infections in cats emphasizes the importance of a regular preventive regimen in this species.

Recent research has shown that heat treating serum samples prior to antigen testing increases the sensitivity of heartworm detection by releasing antigen bound in immune complexes (Little et al., 2014, DiGangi et al., 2017, Gruntmeir et al., 2017, Little et al., 2018). In our population, heat treatment increased antigen test sensitivity in dogs. Two dogs with *D. immitis* microfilariae were antigen negative when non-treated samples were tested, but positive after heat pre-treatment of the serum sample. This agrees with previous studies (Little et al., 2018) and reinforces the importance of routine microfilariae testing when screening dogs for heartworm infection as is recommended by the American Heartworm Society (American Heartworm Society, 2018). In addition, microfilariae of four dogs were identified as *A. reconditum* by morphology and PCR. Half (2/4) of these dogs were antigen-positive prior to heat treatment and all (4/4) were antigen-positive after heat treatment. In a previous multi-center shelter study, six of 616 dogs sampled were positive for *A. reconditum* microfilariae and all were antigen-negative prior to and after heat treatment (DiGangi et al., 2017), a finding consistent with one of the author’s experience in clinical samples (SL). Because they are shelter dogs, we do not know the actual infection status of the four dogs in the present study; these dogs may harbor concurrent *D. immitis* and *A. reconditum* infections, but without detectable *D. immitis* microfilariae. Alternatively, some or all of these antigen tests may be false positives. False positive antigen tests have been reported both before and after heat pre-treatment of serum (Little et al., 2018). One additional dog had circulating *D. repens* microfilariae, a nematode not considered present in the United States other than in dogs with a travel history (Rishniw et al., 2006; Liotta et al., 2013). This dog was antigen-negative prior to heat treatment and antigen-positive post heat treatment. In one study evaluating heat treatment in *D. repens* infected dogs in a *D. immitis*-free area, heat treatment decreased the specificity of HW antigen tests (Venco et al., 2017). It is possible that heat treatment in the present study led to a false-positive HW antigen test or that the dog harbored a concurrent *D. immitis* infection. A total of nine dogs converted from antigen-negative to positive after heat treatment, but did not have detectable microfilariae. These dogs may have had early *D. immitis* infections; heat treatment has been shown to detect experimental infections as early as 4.2 months (Carmichael et al., 2016). The results from this study support those from other studies demonstrating that heat treatment increases antigen test sensitivity, especially in dogs considered at risk of HW infection but with negative in-clinic antigen test results (Little et al., 2018). However, false positive antigen tests can occur both before and after heat pre-treatment of samples (Little et al., 2018). Care should be taken to accurately identify circulating microfilariae in infected dogs and cats to aid in accurately interpreting antigen test results. Heat pre-treatment of samples was not included in the USDA approval of heartworm tests, and any test result should be incorporated into the overall clinical evaluation of a given patient before making a decision about infection status and appropriate treatment.

The prevalence of HW antigen in our shelter cat population was 3% regardless of whether heat treatment was performed. To our knowledge there has been only one other published study measuring HW antigen prevalence in shelter cats using pre and post heat treatment samples. Gruntmeir et al. (2017) evaluated shelter cats from northeastern Oklahoma (*n* = 116) and the southeastern United States (*n* = 104). The prevalence of HW antigen increased from 0.5 to 5.9 % after heat treatment. In that study, heartworm antibody prevalence was significantly higher in cats that were antigen-positive after heat treatment compared to cats that were antigen-negative after heat treatment. In our study the cats that were antigen-positive before and after heat treatment were also antibody-positive by at least one test; however, the one cat that converted from antigen-negative to antigen-positive after heat treatment was negative for antibodies. It is unclear why there is a discrepancy between the pre and post heat-treatment HW antigen prevalence in these two different populations of shelter cats. It is possible that the timing of collection could have affected the proportion of cats sampled with adult heartworm infections in our study. We sampled cats in the months of May and June. In one retrospective study, 23 of 25 of feline heartworm infections in Louisiana were detected from August to December; however, in another retrospective study in North Carolina, the majority of adult heartworm infections were diagnosed from January to September (Guerrero et al., 1992, Atkins et al., 2000). Collecting samples throughout the year in this population of shelter cats could result in sampling more cats with adult infections and thus potentially increase the proportion of cats that convert from antigen-negative to positive after heat treatment.

In this study, two cats had detectable circulating microfilariae; however, they were not *D. immitis* microfilariae. One of these cats harbored large microfilariae but the sequence did not match any available reference sequences and was distinct from that of a *Dirofilaria* sp. sequence amplified from frozen spleen of a bobcat that presumably harbored *D. striata* (data not shown); we suspect this cat was infected with a novel *Dirofilaria* sp. The other cat in our study had *Dirofilaria repens* microfilariae as confirmed by morphology and sequencing and represents the first documented case of *D. repens* in a cat in the United States to our knowledge. Cats and dogs are reservoirs for this zoonotic parasite in Europe, Asia, and Africa (Simón et al., 2012). Because the history of this cat is unknown, it is not possible to know if this cat was infected in the United States or was transported from an endemic country.

This study had limitations that should be considered. The source of dogs and cats in this study was exclusively animal shelter stray intakes over the course of a few weeks, potentially limiting the generalizability to other canine and feline populations. The relatively small sample size could have limited our ability to demonstrate differences between groups or risk factors for infection. All participating animals were admitted to the shelters as strays with no known owners, but it is possible that some were owned pets that had received heartworm preventives. The inability to confirm the true HW infection status, because of the inherent limitations of each of the testing methods coupled with the lack of necropsy data, was another limitation, as these were shelter pets on their way to adoptive homes. To mitigate age as a potential factor influencing HW exposure, all cat and dog pairs were matched within an estimated 2 years, but ages were estimates only. Further, seasonal effects on mosquito transmission could have influenced our results, but our primary aim was to compare dogs and cats, and all specimens were collected in the same month in both species. Regardless, the pathophysiology of heartworm disease in dogs and cats is quite different and even though we used antibody tests to detect HW larval development in cats, because some cats infected with adult worms can become antibody negative, we may be underestimating the true risk of infection when comparing antibody tests in cats to antigen tests in dogs, since dogs with HW infection remain antigen positive for years. On the other end of the spectrum, it is possible that heat treating serum samples prior to antigen testing may have overestimated canine heartworm prevalence. Since the specificity of the antigen test with this sample preparation is not fully characterized, it is possible that some dogs that were truly negative for heartworm infection converted to a false antigen positive post heat treatment.

## 5 Conclusions

This study of stray animals presented to Florida shelters demonstrated similar proportions of cats with evidence of previous or current HW infection and dogs with adult infections. The prevalences of infection in both species from this population were relatively high, emphasizing the need for routine year-round HW preventives in both dogs and cats. Moreover, improved awareness of larval, immature, and adult HW infection prevalence, especially in cats, and more accurate and comprehensive diagnostic techniques for both species, are required to provide optimal supportive care for all pets. Shelters in high prevalence areas should prioritize allocation of resources for heartworm screening and prevention, especially for animals being transported to low prevalence areas of the country, where awareness of HW screening and prevention could be suboptimal.

## Declaration of Competing Interest

Jessica Rodriguez, Deborah Amodie, Kennedy Mwacalimba, and Annette Litster are employed by Zoetis. All authors have seen and approved the final version of the manuscript being submitted. They warrant that the article is the authors' original work, has not received prior publication and is not under consideration for publication elsewhere.

## CRediT authorship contribution statement

**Kellie M. Hays:** Conceptualization, Methodology, Formal analysis, Investigation, Formal analysis, Investigation, Writing - original draft, Writing - review & editing. **Jessica Y. Rodriguez:** Conceptualization, Methodology, Investigation, Writing - original draft, Writing - review & editing, Supervision. **Susan E. Little:** Conceptualization, Methodology, Investigation, Resources, Writing - original draft, Writing - review & editing, Supervision. **Annette L. Litster:** Conceptualization, Methodology, Investigation, Writing - original draft, Writing - review & editing, Supervision. **Kennedy K. Mwacalimba:** Conceptualization, Methodology, Investigation, Resources, Project administration, Funding acquisition. **Kellee D. Sundstrom:** Investigation. **Deborah M. Amodie:** Methodology, Formal analysis, Resources, Writing - original draft. **Maria A. Serrano:** Conceptualization, Investigation, Resources. **Simone D. Guerios:** Investigation, Resources. **Jennifer N. Lane:** Conceptualization, Investigation. **Julie K. Levy:** Conceptualization, Methodology, Formal analysis, Investigation, Resources, Writing - original draft, Writing - review & editing, Supervision, Project administration, Funding acquisition.

## References

[bib0005] American Heartworm Society (2018). Current canine guidelines for the prevention. Diagnosis and Management of Heartworm (*Dirofilaria Immitis*) Infection in Dogs.

[bib0010] Atkins C. (2003). Comparison of results of three commercial heartworm antigen test kits in dogs with low heartworm burdens. J. Am. Vet. Med. Assoc..

[bib0015] Atkins C.E., DeFrancesco T.C., Coats J.R., Sidley J.A., Keene B.W. (2000). Heartworm infection in cats: 50 cases. J. Am. Vet. Med. Assoc..

[bib0020] Berdoulay P., Levy J.K., Snyder P.S., Pegelow M.J., Hooks J.L., Tavares L.M., Gibson N.M., Salute M.E. (2004). Comparison of serological tests for the detection of natural heartworm infection in cats. J. Am. Anim. Hosp. Assoc..

[bib0025] Bowman D.D., Atkins C.E. (2009). Heartworm biology, treatment, and control. Vet. Clin. North Am. Small Anim. Pract..

[bib0030] Carmichael J., McCall J., DiCosty U., Mansour A., Roycroft L. (2016). Evaluation of *Dirofilaria immitis* antigen detection comparing heated and unheated serum in dogs with experimental heartworm infections. Parasit. Vectors.

[bib0035] Courtney C.H., Zeng Q.-Y. (1989). The structure of heartworm populations in dogs and cats in Florida. In: Proceedings of the American Heartworm Symposium.

[bib0040] Courtney C.H., Zeng Q.-Y. (2001). Comparison of heartworm antigen test kit performance in dogs having low heartworm burdens. Vet. Parasitol..

[bib0045] DiGangi B., Dworkin C., Stull J., O’Quin J., Elser M., Marsh A., Groshong L., Wolfson W., Duhon B., Broaddus K., Gingrich E., Swiniarski E., Berliner E. (2017). Impact of heat treatment on *Dirofilaria immitis* antigen detection in shelter dogs. Parasit. Vectors.

[bib0050] Dillon A.R., Blagburn B.L., Tillson M., Brawner W., Welles B., Johnson C., Cattley R., Rynders P., Barney S. (2017). The progression of heartworm associated respiratory disease (HARD) in SPF cats 18 months after *Dirofilaria immitis* infection. Parasit. Vectors.

[bib0055] Drake J., Parrish R.S. (2019). Dog importation and changes in heartworm prevalence in Colorado 2013- 2017. Parasit. Vectors.

[bib0060] Drake J., Gruntmeir J., Merritt H., Allen L., Little S.E. (2015). False negative antigen tests in dogs infected with heartworm and placed on macrocyclic lactone preventives. Parasit. Vectors.

[bib0065] Goodwin J.K. (1998). The serologic diagnosis of heartworm infection in dogs and cats. Clin. Tech. Small Anim. Pract..

[bib0070] Gruntmeir J., Adolph C., Thomas J., Reichard M., Blagburn B., Little S. (2017). Increased detection of *Dirofilaria immitis* antigen in cats after heat pretreatment of samples. J. Feline Med. Surg..

[bib0075] Guerrero J., McCall J.W., Dzimianski M.T., McTier T.L., Holmes R.A., Newcomb K.M., Soll M.D. (1992). Prevalence of *Dirofilaria immitis* Infection in Cats from the Southeastern United States.

[bib0080] Kaiser L., Williams J. (2004). *Dirofilaria immitis:* worm burden and pulmonary artery proliferation in dogs from Michigan (United States). Vet. Parasitol..

[bib0085] Ledesma N., Harrington L. (2011). Mosquito vectors of dog heartworm in the United States: vector status and factors influencing transmission efficiency. Top. Companion Anim. Med..

[bib0090] Lee A., Atkins C. (2010). Understanding feline heartworm infection: disease, diagnosis, and treatment. Top. Companion Anim. Med..

[bib0095] Levy J., Snyder P., Taveres L., Hooks J., Pegelow M., Slater M., Hughes K., Salute M. (2003). Prevalence and risk factors for heartworm infection in cats from northern Florida. J. Am. Anim. Hosp. Assoc..

[bib0100] Levy J.K., Burling A.N., Crandall M.M., Tucker S.J., Wood E.G., Foster J.D. (2017). Seroprevalence of heartworm infection, risk factors for seropositivity, and frequency of prescribing heartworm preventives for cats in the United States and Canada. J. Am. Vet. Med. Assoc..

[bib0105] Liotta J.L., Sandhu G.K., Rishniw M., Bowman D.D. (2013). Differentiation of the microfilariae of *Dirofilaria immitis* and *Dirofilaria repens* in stained blood films. J. Parasitol..

[bib0110] Litster A., Atkins C., Atwell R. (2008). Acute death in heartworm-infected cats: unravelling the puzzle. Vet. Parasitol..

[bib0115] Little S., Raymond M., Thomas J., Gruntmeir J., Hostetler J., Meinkoth J., Blagburn B. (2014). Heat treatment prior to testing allows detection of antigen of *Dirofilaria immitis* in feline serum. Parasit. Vectors.

[bib0120] Little S., Saleh M., Wohltjen M., Nagamori Y. (2018). Prime detection of *Dirofilaria immitis*: understanding the influence of blocked antigen on heartworm test performance. Parasit. Vectors.

[bib0125] Montoya-Alonso J.A., Carretón E., Corbera J.A., Juste M.C., Mellado I., Morchón R., Simón F. (2011). Current prevalence of *Dirofilaria immitis* in dogs, cats and humans from the island of Gran Canaria, Spain. Vet. Parasitol..

[bib0130] NCBI National Center for Biotechnology Information (2019). GenBank Basic Local Alignment Search Tool. https://blast.ncbi.nlm.nih.gov/Blast.cgi.

[bib0135] Otranto D., Diniz D.G., Dantas-Torres F., Casiraghi M., de Almeida I.N., de Almeida L.N., dos Santos J.N., Furtado A.P., de Almeida Sobrinho E.F., Bain O. (2011). Human intraocular filariasis caused by *Dirofilaria* sp. nematode. Brazil. Emerg. Infect. Dis..

[bib0140] Rishniw M., Barr S.C., Simpson K.W., Frongillo M.F., Franz M., Dominguez Alpizar J.L. (2006). Discrimination between six species of canine microfilariae by a single polymerase chain reaction. Vet. Parasitol..

[bib0145] Self S.W., Pulaski C.N., McMahan C.S., Brown D.A., Yabsley M.J., Gettings J.R. (2019). Regional and local temporal trends in the prevalence of canine heartworm infection in the contiguous United States: 2012-2018. Parasit. Vectors.

[bib0150] Simón F., Siles-Lucas M., Morchón R., González-Miguel J., Mellado I., Carretón E., Montoya-Alonso J.A. (2012). Human and animal dirofilariasis: the emergence of a zoonotic mosaic. Clin. Micro. Rev..

[bib0155] Snyder P.S., Levy J.K., Salute M.E., Gorman S.P., Kubilis P.S., Smail P.W., George L.L. (2000). Performance of serologic tests used to detect heartworm infection in cats. J. Am. Vet. Med. Assoc..

[bib0160] Strickland K.N. (1998). Canine and feline caval syndrome. Clin. Tech. Small Anim. Pract..

[bib0165] Tzipory N., Crawford P.C., Levy J.K. (2010). Prevalence of *Dirofilaria immitis*, *Ehrlichia canis*, and *Borrelia burgdorferi* in pet dogs, racing greyhounds, and shelter dogs in Florida. Vet. Parasitol..

[bib0170] VassarStats: Statistical Computation Web Site. www.VassarStats.com (Accessed 15 November 2019).

[bib0175] Venco L., Genchi M., Genchi C., Gatti C., Kramer L. (2011). Can heartworm prevalence in dogs be used as provisional data for assessing the prevalence of the infection in cats?. Vet. Parasitol..

[bib0180] Venco L., Manzocchi S., Genchi M., Kramer L.H. (2017). Heat treatment and false-positive heartworm antigen testing in ex vivo parasites and dogs naturally infected by *Dirofilaria repens* and *Angiostrongylus vasorum*. Parasit. Vectors.

[bib0185] Vieira L., Sivestre-Ferreira A.C., Fontes-Sousa A.P., Balreira A.C., Morchón R., Carretón E., Vilhena H., Simón F., Montoya-Alonso J.A. (2014). Seroprevalence of heartworm (*Dirofilaria immitis*) in feline and canine hosts from central and northern Portugal. J. Helminthol..

[bib0190] Winter R., Dillon A., Cattley R., Blagburn B., Tillson D., Johnson C., Brawner W., Welles E., Barney S. (2017). Effect of heartworm disease and heartworm-associated respiratory disease (HARD) on the right ventricle of cats. Parasit. Vectors.

[bib0195] Zajac A.M., Conboy G.A. (2012). Veterinary Clinical Parasitology.

